# EDUCATIONAL OUTREACH IN ALZHEIMER’S DISEASE AMONG OLDER AFRICAN AMERICANS

**DOI:** 10.1093/geroni/igz038.3166

**Published:** 2019-11-08

**Authors:** Ashley R Shaw, Briana Bright, Jaime Perales Puchalt, Eric Vidoni, Gabriela Amparan, Broderick Crawford

**Affiliations:** 1 University of Kansas Alzheimer’s Disease Center, Fairway, Kansas, United States; 2 University of Kansas Medical Center, Kansas City, Kansas, United States; 4 NBC Community Development Corporation, Kansas City, Kansas, United States

## Abstract

Alzheimer’s disease (AD) is a growing public health problem that continues to disproportionally impact African Americans. African Americans are twice as likely to be afflicted with AD compared to non-Latino Whites. However, continued lack of inclusion of African Americans in clinical research trials may reduce the generalizability of future treatments. We investigated how culturally tailored prevention education impacted knowledge, attitudes, and beliefs of AD among older African Americans. We also assessed how culturally tailored prevention education impacted participation in clinical research trials among older African Americans. Researchers delivered “Aging with Grace,” a culturally tailored dementia program to community and faith-based organizations. Demographic information, knowledge of AD, and beliefs of clinical research trials were collected using pre- and post-surveys. In addition, information from community members interested in enrolling in a clinical research study was acquired. A total of 66 community members attended “Aging with Grace” from March to August 2019. 32% of participants perceived an increase in AD knowledge. Most participants (89.1%) believed that more African Americans should participate in research and 29 (44%) expressed interest in enrolling in clinical trials (observational – 73.2%, prevention – 68.2%, treatment – 24.4%). Most participants (93.1%) rated the presentation highly informative and 78% reported that the presentation was very applicable to their daily life. Overall knowledge of AD and interest in participating clinical trials improved with culturally tailored education. Future research should explore ways of enhancing knowledge and participation to enhance inclusion in prevention and treatment trials.

